# Photodynamic Therapy Targeting Matrix Metalloproteases in Cancer: Standpoint for an Innovative Anticancer Strategy

**DOI:** 10.3390/cimb48050441

**Published:** 2026-04-23

**Authors:** Vanya Mantareva, Diana Braikova, Mingna Sun, Jianye Zhang

**Affiliations:** 1 Institute of Organic Chemistry with Centre of Phytochemistry, Bulgarian Academy of Sciences, Acad. G. Bonchev, Str. Bld. 9, 1113 Sofia, Bulgaria; 2 Guangzhou Municipal and Guangdong Provincial Key Laboratory of Molecular Target & Clinical Pharmacology, The NMPA and State Key Laboratory of Respiratory Disease, The Fifth Affiliated Hospital and School of Pharmaceutical Sciences, Guangzhou Medical University, Guangzhou 511436, China; mingnas85@163.com (M.S.);; 3 The Affiliated Traditional Chinese Medicine Hospital, Guangzhou Medical University, Guangzhou 510000, China

**Keywords:** photodynamic therapy, matrix metalloproteases, inhibitors, photosensitizers, cancer, metastases, anticancer therapeutic strategy

## Abstract

Matrix metalloproteases (MMPs), which are activated during malignancy growth and metastasis, have been a focus of current anticancer research and development. The selective recognition and precise inhibition of active MMPs could limit cancer progression. Photodynamic therapy (PDT) is a well-established local treatment for solid tumors. MMPs are expressed primarily in the vicinity of the tumor, and PDT strongly influences this process. However, in rare cases, PDT can activate MMPs. An improved PDT outcome was observed with the action of an MMP inhibitor (MMPI), namely Prinomastat. Research on this topic remains limited, presenting a substantial opportunity for further investigations. Various small-molecule compounds have been designed to target MMPs as potential inhibitors, but clinical trials have not confirmed their efficacy in vitro or in vivo. Currently, novel MMPIs with complex chemical structures are being designed and have shown high efficacy in initial preclinical studies. This review aims to provide a critical overview of recent advances in the use of cancer-related MMPs as therapeutic targets, along with new innovative approaches to targeting them. The effects of PDT on cancer-related MMPs, along with the advantages of combining therapies that could enhance curative efficacy, are discussed. The novel inhibitory approaches that regulate cancer-related MMPs are summarized.

## 1. Introduction

Proteolytic enzymes are large molecules with vital roles in all living processes [1]. These enzymes are highly important in biochemistry, participate in many essential biological reactions, and can cause life-threatening diseases in humans when homeostasis is disrupted. Additionally, they begin breaking down proteins by hydrolyzing the amide bonds between amino acids. Proteolytic enzymes are categorized based on the amino acids in their catalytic centers, including metalloproteases, as well as aspartic, cysteine, serine, glutamic, and threonine proteases [2].

Dysregulation of the extracellular matrix (ECM) is known to trigger cell proliferation, invasion, and tissue fibrosis [3]. It is well documented that matrix metalloproteases (MMPs) influence carcinogenesis and ECM degradation, serving as the initial stage of tumor cell invasion, which damages the cell membrane and the surrounding stroma containing fibrillar collagens [4]. Additionally, MMPs play a role in angiogenesis, promoting the growth, migration, and metastasis of cancer cells [5]. Their biological activity is also regulated by a family of native tissue inhibitors (TIMPs). An imbalance between MMPs and TIMPs leads to harmful tissue remodeling processes, especially during cancer development and spread [6]. TIMPs are known to have positive effects on healthy cells and are among the native inhibitors of MMPs that circulate in body fluids and across cells and tissues [7]. Moreover, TIMPs are key regulators of MMPs, which degrade the ECM while maintaining cell-surface biomolecules. Research on TIMP–MMP complexes has clarified the mechanisms by which TIMPs inhibit MMPs [7,8]. The numerous interaction sites with enzymes enable TIMPs to inhibit MMPs effectively and selectively [9]. Developing TIMP alternatives is complicated because they may exhibit different molecular behaviors due to interactions with proteases. On the other hand, TIMPs also have functions independent of MMPs [10]. Their actions involve cell growth and differentiation, migration, antiangiogenic effects, apoptosis, and synaptic plasticity.

Specific protease activation in cancer cells has emphasized MMPs as vital diagnostic and therapeutic biomarkers. Cells with dysregulated MMP activity exhibit changes that lead to abnormal proteolysis and disease states [11]. Receptors responsible for these activities have been identified, and their signaling pathways studied to support new inhibition strategies. The extensive preclinical data across various cancers suggest that inhibiting MMPs can be an effective therapy [12]. MMP inhibitors (MMPIs) remain a key focus of research to develop more effective anticancer drugs. Although initial animal studies showed promise, early clinical trials were not very encouraging regarding the effectiveness of MMPIs alone [13]. The development of targeted, specific inhibitors for individual MMPs remains an active area of research. This involves designing drugs that identify chemical structures with high binding affinity, favorable pharmacokinetics, and selective inhibition of MMPs involved in tumor growth [14]. Likely, the failures seen in clinical trials are linked to poor trial designs, unrealistic inhibitor protocols, and the complexity of various cancer-related MMPs [15]. Many other differences between the studied in vivo models and human clinical trials still need to be addressed.

Photodynamic therapy (PDT) is a superficial, local method with several advantages over other cancer treatments [16]. PDT overcomes the main limitations of chemical inhibitors, such as toxicity and lack of specificity, which often cause damage to healthy tissue. Innovative cancer technologies could enhance the activity of a photosensitizer (PS) against targets such as cancer-related MMPs, especially when combined with other anticancer methods. The primary goals of newly developed anticancer drugs and treatment protocols are to overcome limitations in drug delivery, toxicity, selectivity, tumor penetration, uptake, tissue and organ retention, and clearance.

The review aims to present an actual overview of existing knowledge on the main cancer-related MMPs as therapeutic targets. It highlights the PDT method and recent small-molecule inhibitors, which, when combined, can enhance treatment effectiveness. Based on early clinical and current experimental studies, the structures and functions of effective MMPIs are summarized. Available PDT research on MMPs has been conducted both in vitro and in vivo, suggesting that PDT affects MMP activity. The research on regulating them through combined therapy is thoroughly reviewed. Perspectives on using PDT to specifically target cancer-related MMPs during early cancer growth and to influence multiple inhibitory mechanisms for an effective anticancer strategy are discussed.

## 2. Matrix Metalloproteases Related to Cancer

**Figure 1:**
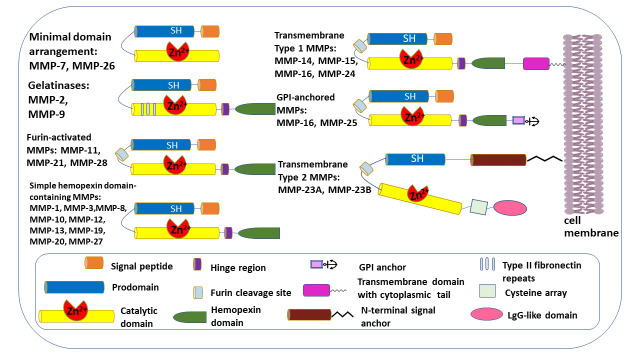
**Domain structure of matrix metalloproteases.** MMPs consist of a prodomain, an N-terminal signal peptide, and a catalytic domain. A cysteine (SH) switch in the prodomain forms a complex with catalytic zinc (Zn^2+^) in the catalytic domain, blocking enzymatic activity. Most MMPs also have a linker (hinge region) and a hemopexin domain at the C-terminus of the catalytic domain. MMP-2 and MMP-9 have three repeats of a type II fibronectin domain inserted into the catalytic domain, which bind to gelatin, collagens, and laminin. All transmembrane MMPs and glycosylphosphatidylinositol (GPI)-anchored MMPs feature a furin cleavage site at the C-terminal end of their prodomains. MMP-16 and MMP-25 are anchored to the plasma membrane via a GPI anchor. MMP-14, MMP-15, MMP-16, and MMP-24 are anchored to the cell membrane through transmembrane domains with cytoplasmic tails. A cysteine array at the C-terminal of MMP-23 and an immunoglobulin (IgG)-like domain occupy the hemopexin domain.

Significantly increased MMP activity in various cancers, which is linked to poorer prognosis and lower survival rates, can be determined in the tumor microenvironment (TME). Studies have shown that a key factor in the development and spread of malignant melanoma is a family of MMPs, including MMP-1, -2, -3, -9, -13, and MMP-14 [17,18]. The ECM has become a primary driver of tissue remodeling, particularly via MMPs such as gelatinases. Currently, emerging technologies are enhancing understanding of the relationships between specific proteases and their substrates, providing insights into their pathological effects [18]. The process is complex, involving interconnected proteases, substrates, regulators, and effectors that must work together to achieve effective therapeutic results. Therefore, precise regulation of proteases is crucial to prevent misdirected proteolytic activity. MMPs promote cancer development by cleaving growth factors, degrading the ECM, and regulating receptor cleavage, thus disrupting migratory signals [19]. MMPs play a dual role, potentially through interactions with different signaling pathways, by promoting tumor growth through degradation of extracellular matrix barriers and by inducing angiogenesis [20]. MMPs have distinct substrate specificities that sometimes overlap, and an alternative classification is based on their domain organization (Figure 1).

Studies suggested that proteases involved in tumor growth and progression depend on nutrient and oxygen availability [21]. Components of the TME are crucial to tumor growth and spread, so therapies targeting the TME must act through multiple mechanisms [22]. These include altering hypoxia, controlling blood vessels, and reprogramming immune cells. In this context, oxygen-dependent PSs for PDT appear to interact effectively with MMPs [23]. PDT effectiveness could be realized through dual MMP-related action, which can be achieved by manipulating the TME [24]. The benefits of PDT can be maximized by balanced protocols that optimize photosensitizer features, light settings (fluence, dose, and spectrum), and oxygen levels. To maintain a critical balance between promoting and inhibiting tumor growth during treatment, drug delivery, retention, and absorption in cancer tissues should be selective [25]. Appropriate light parameters, such as using a far-red spectrum that allows deeper light penetration and administering low-dose, fractionated irradiation, are suggested to reduce rapid vascular damage and avoid immune suppression in tumor cells [26]. The high levels of reactive oxygen species (ROS) produced during photosensitization, although damaging to normal cells, can also promote tumor growth and can be regulated by an appropriate light protocol. Conversely, the hypoxic TME may support tumor progression by stimulating growth factor production. Known methods to improve oxygenation include oxygen-generating compounds, perfluorocarbon carriers for direct oxygen delivery, and inhibitors such as HIF-1α that block pro-tumor pathways triggered by hypoxia [27]. During tumor progression, the combined dual action of PDT and these factors can help suppress tumor cell growth. According to an early study, zinc(II) ions play a crucial role in the catalytic and structural functions of metalloenzymes, making them therapeutic targets [28].

**Figure 2:**
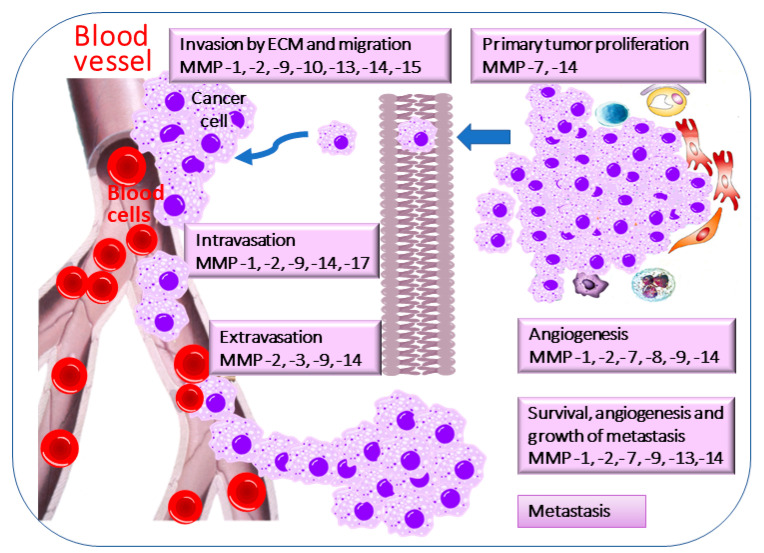
**Tumor and metastasis development, and matrix metalloproteases related to these processes.** Metastasis is a highly complex process that remains a major obstacle to curative treatment. It involves changes in the tumor microenvironment that affect blood flow, increase blood vessel pressure, facilitate adaptation to new cellular conditions at secondary sites, and help prevent lethal immune cell attacks. MMPs are involved in many stages of tumor metastasis. The five key stages of metastatic development are invasion, intravasation, circulation, extravasation, and colonization. Consequently, tumor cells cannot successfully metastasize without MMPs.

The ability of tumor cells to detach from a primary tumor mass, migrate through the body, and eventually form secondary tumors is generally considered the foundation of metastasis [29]. The specific mechanism underlying MMP-mediated cancer development is shown in Figure 2. The progression of metastasis, which requires cancer cells to leave their primary site and then enter blood or lymphatic vessels, is known as intravasation [30]. It is facilitated by interactions with vessel walls and potentially by remodeling the vessels themselves [31]. Typically, in cancer, inflammation, repair, and other remodeling processes involve matrix turnover, cell migration, and the processing of secreted factors, all of which are mediated by MMPs [32]. Collagenase-1 and -3, along with stromelysins-1, -2, and -3, are either absent in healthy cells or exhibit undetectable proteolytic activity [33]. In contrast, in cancerous tissues, the levels of these active MMPs are higher. It is believed that a clear understanding of the mechanisms behind this multistep process could greatly assist in developing a therapy to prevent metastasis [34]. Several models have been developed to better understand tumor colonization, spread, and metastatic progression [35]. Dysregulation of MMPs tends to accelerate cancer growth and spread, helping tumor cells evade immune detection and destabilize the stroma.

Cancer cells typically resist oxidative stress by renewing their metabolic pathways to boost antioxidant capacity. Evidence suggests that increased oxidative stress may promote the spread of tumor cells to other organs [36]. This has been observed across many cancer types and has to be a key focus in metastasis research. After extravasation, metastatic cells must adapt to diverse metabolic environments in target organs and to hypoxia resulting from the lack of blood vessels. The vasculature deficiency also subjects circulating cancer cells to nutrient stress [37]. MMPs’ roles in tumor invasion and metastasis have been described, including their enzymatic function in ECM remodeling, as reported by Parks and co-workers [38]. This includes effects on cellular and molecular signaling, immune system interactions, regulatory mechanisms, and potential therapeutic applications. MMP-1 supports cancer cell invasion and lung metastasis during tumor progression by degrading the ECM. It is produced not only by cancer cells but also by stromal and inflammation-related cells [39]. However, the specific role of MMP-1 expression in host cells, especially tumor-associated macrophages (TAMs) and lung parenchymal cells, remains unclear [40]. As noted earlier, MMPs are valuable targets for treating many diseases [41]. For example, the complex interactions in colorectal cancer development emphasize the importance of MMPs, particularly gelatinases, as mediators of disease progression [42]. The gelatinases, MMP-2 and MMP-9, have been shown to significantly influence colorectal cancer progression, invasion, and metastasis, and, in fibrosarcoma, they support tumor growth, invasion, and metastasis [43]. These MMPs are also promising therapeutic targets. The targeted applicability across various malignancies was determined by a comprehensive examination of the occurrence of all MMP subtypes at the genetic, protein, and activity levels in both healthy and diseased tissues.

Disruption of the physiological balance between MMP activation and deactivation could lead to disease progression [44]. The biochemical and clinical studies on MMPs in young patients suggest their relevance for diagnosis, prognostication, and monitoring therapeutic effects [45]. MMP-11, stromelysin-3, is a member of the MMP superfamily that has been detected in cancer cells, stromal cells, and the surrounding environment [46]. In the early stages, MMP-11 overexpression has been shown to promote carcinogenesis. In later stages, however, tumor cell spread is inhibited. Potential biomarkers for early cancer diagnosis, prognosis, and treatment monitoring were identified, including tissue and serum samples from patients with cervical cancer, which exhibit significantly higher MMP-7 levels than those from healthy individuals, signifying MMP-7 as a useful biomarker [47]. Table 1 presents a summary of MMP classes in relation to cancer and metastases. It summarizes MMP groups into several well-recognized subfamilies based on substrate specificity and defines their roles at various stages of cancer development and metastasis. The table may be useful for identifying the future clinical significance of MMPs, including their roles as prognostic biomarkers, in early detection, and as targets for therapeutic intervention. In several malignancies, including colorectal and breast cancer, elevated levels of specific MMPs, such as MMP-7 and MMP-9, are frequently linked to poor prognosis and increased metastatic potential. As can be seen, a high expression of MMP-2, -7, and -9 is often associated with more aggressive tumors and shorter overall survival in cancers such as gastric, breast, and colorectal. Table 1 also highlights less-studied enzymes, such as MMP-26, as a potential new target in early skin carcinogenesis. Identifying which MMP subfamilies are overexpressed in a specific tumor will aid in selecting more targeted therapies. For instance, this could guide the development of more selective inhibitors rather than broad-spectrum ones that have proven ineffective in clinical trials. Research indicates that some MMPs (such as MMP-8 and MMP-12) may have tumor-suppressive effects in specific contexts (e.g., skin, breast, and colorectal cancers). This suggests that they should not be inhibited during therapy. The table clarifies how different MMPs contribute to cancer spread and metastasis. It highlights which enzymes (for example, MMP-13 and MMP-14) are responsible for creating new blood vessels that support malignancies. In summary, the main oncology benefits of the information in Table 1 are diagnostic, such as identifying MMPs in blood or tissue clinical samples for early cancer diagnosis. Linking high MMP levels to the risk of recurrence and shorter survival is also of prognostic value.

## 3. Synthetic Small-Molecule Inhibitors

The small-molecule inhibitors are still under development and show promise in inhibiting MMPs in “in vitro” and “in vivo” studies [129]. Their mechanisms of action include disrupting essential pathways involved in tumor growth and cell invasion (Figure 2). Additionally, they have a crucial role in tumor angiogenesis and the development of various cancers. However, current small-molecule inhibitors have unsatisfactory outcomes in clinical trials due to their broad, nonspecific activity and side effects [130].

Developing selective inhibitors with distinct mechanisms of action against specific MMPs remains challenging. An enzyme-triggered multistage platform was recently developed to enhance the targeted delivery of medications to tumor cells [131]. This platform features a porous silicon microparticle linked to drug-loaded polymeric nanoparticles. The study demonstrated that increased nanoparticle uptake into cancer cells via an enzyme-activated strategy promotes their release into the tumor. When the nanoparticles encounter MMP-2 in the tumor, the linker between them can be cleaved, allowing them to enter cancer cells. Both in vitro and in vivo studies showed that this enzyme-responsive approach increased the uptake of the load by the tested melanoma cells. One way to maintain healthy MMP levels is to consume natural inhibitors found in polyphenol-rich foods [132]. A novel MMP-targeting approach involves natural hydrolytic activity and the resulting catalytic signal amplification by proteinases. Furthermore, flavonoids and other plant-derived isolates are among the next-generation inhibitors of MMPs [133]. Enzymatic chemical processes using nanoparticles can activate anticancer effects in three dimensions and over time, enhance the corresponding response, and mechanically damage cancer cells [134].

A relatively large number of MMPs have been identified as therapeutic targets in cancer [135]. Their active forms are associated with cancer growth and metastasis progression. The results showed that pulmonary fibrosis, a precancerous condition, is mediated by MMP-7 expression [136]. Intratracheal bleomycin therapy does not cause pulmonary fibrosis in MMP-7 knockout (KO) mice. Based on these findings, matrilysin modulates pulmonary fibrosis and could be a target for inhibitory therapy. Matrilysin (MMP-7) is produced by glandular epithelial cells and macrophages [137]. MMP-7 is overexpressed in human cancer cells associated with esophageal, cholangiocarcinoma, gastric, colon, prostate, and bladder cancers. Another study found that breast cancer tissue has higher levels of MMP-1 and MMP-3 than normal breast epithelial tissue [138]. Blocking these MMPs might help prevent early-stage breast cancer and reduce the spread of existing tumors [139]. More research is needed to understand the distinct roles of MMPs across cancer types fully and to develop reliable, clinically useful MMP-based diagnostic and prognostic tools. Given the high diversity of MMP subtypes, the specific expression profiles associated with particular malignancies were identified [140]. This underscores the potential for tumor detection and the development of selective drugs to prevent cancer progression.

**Figure 3:**
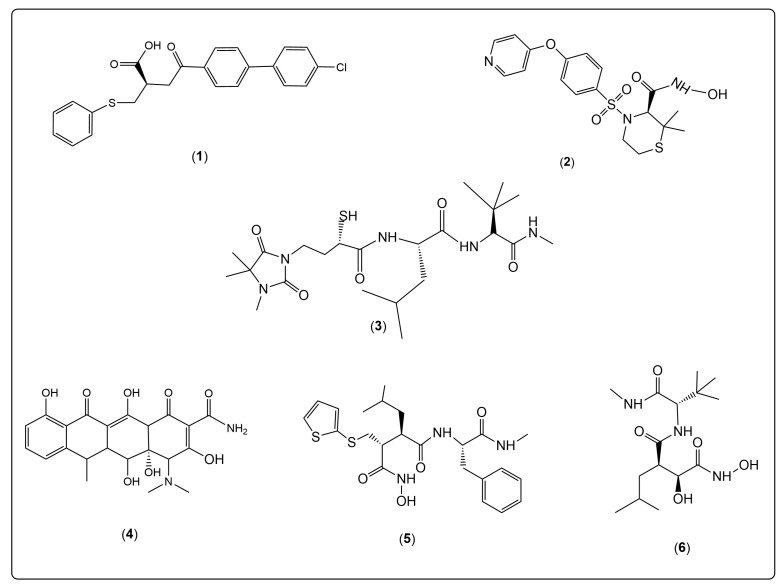
**Matrix metalloprotease inhibitors at preclinical (1–4) and clinical (5 and 6) stages.** They include tanomastat (1), prinomastat (2), rebimastat (3), and doxycycline hyclate (4). The peptidomimetic inhibitors are batimastat (5) and its analog, marimastat (6), which bind to the active-site zinc ion through a covalent bond.

Recently, several inhibitory drugs have been developed to target MMP enzymatic activity [141]. The first generation of MMPIs includes various peptide-based structures [142]. The second group features nonpeptide inhibitors that target specific aspects of the MMP active site and were designed to improve the pharmacological properties and oral absorption of peptides [143]. However, clinical trials with both generations of MMPIs often fail to produce the expected results. The research and development of MMPIs remains a significant challenge. Current studies investigate the properties of new MMPIs in relation to cancer therapy [144]. Among the new generation small-molecule inhibitors are the compounds shown in Figure 3. These MMPIs include tanomastat (1) and prinomastat (2) [145], which appear to be improved versions of earlier similar-structured inhibitors. The third compound, rebimastat (3), inhibits multiple MMPs (MMP-1, -2, -3, -8, -9, -13, and -14) and has demonstrated effectiveness in treating breast, prostate, lung, ovarian, and pancreatic cancers [146]. This compound was withdrawn from clinical trials. Early peptidomimetic inhibitors, such as batimastat (5) and its analog marimastat (6), bind to the active-site zinc ion via a covalent bond [147].

Doxycycline hyclate (4), a classic tetracycline antimicrobial drug, has been studied for its potential in cancer treatment and exhibits promising MMP-inhibitory activity [148]. The carboxylate group shows better MMP-2 selectivity for the zinc ion’s catalytic center than other compounds [149]. It has demonstrated effective antiproliferative activity against a wide range of tumors after oral and intraperitoneal administration. The non-antibiotic, chemically modified tetracyclines have been shown to inhibit MMPs and reduce tumor growth and metastasis in animal studies [150]. These findings highlight the continued importance of exploring novel inhibitors. The triple-helical transition-state analog, a new class of protease inhibitors, maximizes selectivity by interacting with both prime and non-prime active-site subsites and secondary binding sites [151]. It demonstrates high affinity and selectivity for gelatinases (MMP-2 and MMP-9).

The rapid acceptance of drugs against cancer is inappropriate, given the non-confirmatory clinical results [152]. A new generation of MMPIs with improved selectivity and pharmacokinetics has been developed, resulting in better toxicity profiles [153]. Several synthetic MMP-specific inhibitors have been created as anticancer agents, as outlined in a review by Fisher et al. [154]. Focusing on novel chemical structures with high affinity and selectivity, the study examined the structural evolution of small-molecule inhibitors.

## 4. Photodynamic Therapy and Matrix Metalloproteases

**Figure 4:**
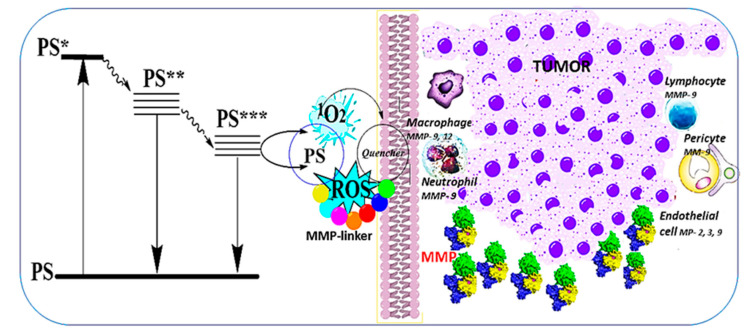
**Cancer-associated matrix metalloproteases as targets in the photodynamic process.** A photosensitizer (PS) is excited at its absorption maximum, thereby becoming a singlet-excited-state molecule (PS*). It then relaxes to a lower-energy-state molecule (PS**), which loses energy via fluorescence. In parallel, its lower-energy triplet-state molecule (PS***) undergoes photosensitization, generating singlet oxygen (^1^O_2_) or other reactive oxygen species (ROS). The target biomolecules are their quenchers that could be affected by these reactive species.

For nearly thirty years, clinical cancer therapy has included PDT as a local, effective curative method [155]. PDT relies on photochemical processes involving long-wavelength-absorbing photoactive molecules, called photosensitizers (PSs), and specific light irradiation in an oxygen atmosphere. Today, PDT is an emerging alternative approach that induces a rapid post-treatment response by affecting MMPs. PDT is characterized by low invasiveness and minimal side effects, with few exceptions. The schematic presentation of the PDT action is illustrated in Figure 4. It begins with light excitation of a photosensitizer (PS), which transitions to its short-lived singlet excited state (PS*), then moves to its forbidden electronic state (PS**), and subsequently converts into a triplet excited-state molecule (PS***). It further reacts via two mechanisms involving electron transfer to surrounding biomolecules (Type I), thereby generating photoinduced reactive oxygen species (ROS). Another pathway (Type II) is known as more productive and involves energy transfer to oxygen, generating reactive, cytotoxic singlet oxygen (^1^O_2_). The formed ROS can be quenched by surrounding biomolecules and the physiological medium (Quencher). Cellular membranes and cancer-related MMPs are also targets in the photosensitization reactions. Typically, tumor cells are surrounded by a stromal compartment composed of fibroblasts, myofibroblasts, vascular endothelial cells, pericytes, macrophages, lymphocytes, and neutrophils (Figure 4). MMPs are expressed not only by tumor cells but also by neighboring host stromal and inflammatory cells in response to factors released by the tumors.

**Figure 5:**
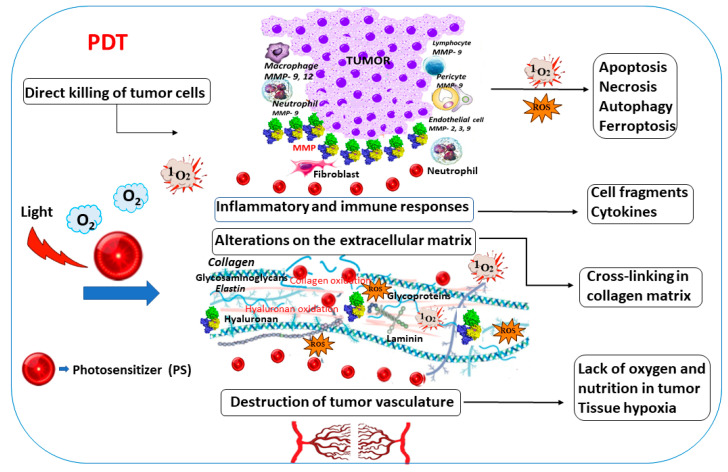
**Impact of photodynamic therapy (PDT) on the tumor and its environment, along with mechanisms of cell death.** Reactive oxygen species (ROSs) destroy tumors when a photosensitizer (PS) accumulates in tumor cells and is subsequently exposed to light. This process involves directly killing tumor cells via apoptosis, necrosis, autophagy, or ferroptosis; cross-linking and other alterations of the collagen matrix; and PDT-induced damage to endothelial cells. These lead to the destruction of the tumor microvasculature and capillaries, resulting in hypoxia, triggering acute inflammation, and inducing an immune response characterized by leukocyte activation and the production of inflammatory mediators, including cytokines, growth factors, and immunoregulators.

PDT-induced irritation leads to infiltration by fibroblasts, macrophages, and MMP-secreting cells, which are involved in ECM degradation, thereby enabling cell migration through the adjacent stroma and regulating receptor cleavage to terminate signaling [156]. The latter further helps the invasion and metastasis of cancer cells. Conversely, MMPs can also inhibit tumor vascularization, exerting a positive influence. A combination of chemo- or immunotherapeutic agents, PDT, and an inhibitor in a multimodal nanoplatform was reported [157]. In some cases, PDT influences MMP expression and activity, and MMPs, in turn, can enhance the therapeutic effect [158]. The mechanisms of cell death involve generating high levels of ROS near cancer cells, thereby increasing ROS accumulation, penetration depth, and the effectiveness of anticancer treatments (Figure 5).

Further research is needed to develop innovative strategies for MMP-targeted PDT, thereby enhancing its efficacy and reducing potential side effects. The impact of photosensitization on MMPs was found to be complex and influenced by multiple factors, which were described early [159]. The PDT method was shown to minimize potential adverse effects on the immune system by precisely activating MMPs, thereby conferring therapeutic efficacy [160]. Both PDT mechanisms involve oxygen and the generation of ROS, which accelerate the transcription of MMPs and other biomolecules. Nelson et al. [161] observed MMP expression and their further activation by oxidative processes, including ROS, in aerobic organisms, mediated by cellular modulation and mitochondrial mechanisms. Liu et al. [162] worked with microarray analysis to determine differences in gene expression between untreated cultured human gingival carcinoma cells (Ca9-22) and identical cells treated with PDT. These results indicated increased MMP-10 mRNA levels, suggesting a cellular self-defense mechanism against PDT-induced damage. These findings indicate that PDT influences MMPs while simultaneously suppressing their overexpression.

**Figure 6:**
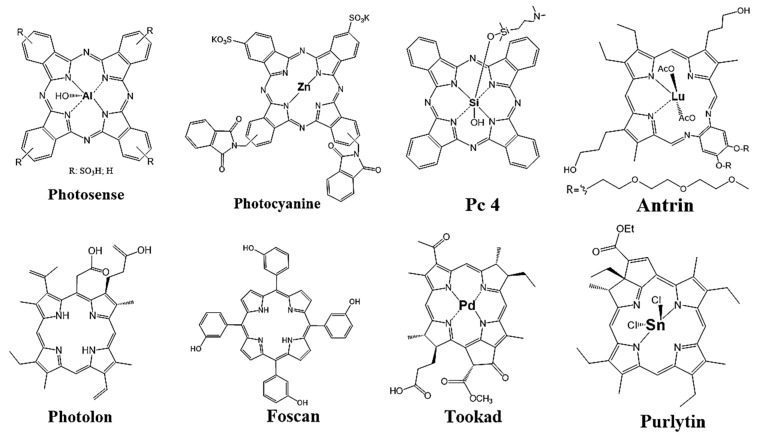
**Porphyrin-type photosensitizers approved for clinical photodynamic therapy**. The phthalocyanines approved for clinical use are Photosense, Photocyanine, and Pc4. A variety of other porphyrin-like structured drugs, including Antrin, Photolon, Foscan, Tookad, and Purlytin, have been evaluated for clinical application in cancer PDT.

The first-generation PDT drugs included hematoporphyrin derivative (HpD) and its purified form, “Photofrin,” both of which received clinical approval in the late 20th century for cancer therapy [163]. Other porphyrin-like drugs, including Antrin, Photolon, Foscan, Tookad, and Purlytin, have also been used for cancer PDT [164]. The drugs for clinical use, such as Photosense, Photocyanine, and Pc4, feature a phthalocyanine core (Figure 6). A recent study reported that the relationship between MMPs and ALA-PDT remains unclear [165]. However, it may be crucial to the physiology of oral cancer and precancerous lesions. Tissue inhibitor of MMP-1 expression decreased, and extracellular MMP-1 inducer increased, as shown in in vivo studies and immunoblot assays after PDT. Gelatin zymography and enzyme activity assays on protein extracts from treated tumors confirmed the presence of both latent and active forms of MMP-9. A study showed that administration of Prinomastat, an MMP inhibitor, resulted in a significant improvement in tumor response to PDT without affecting normal skin photosensitivity [166]. Immunohistochemical analysis indicated that MMP-9 expression after PDT was predominantly in infiltrating inflammatory and endothelial cells, whereas tumor cells showed minimal expression [167]. It was shown that MMP-9 downregulation following PDT with hypericin leads to photoactivation in Epstein–Barr virus-infected human nasopharyngeal cancer cells [168]. Hypericin also inhibited endothelial cell migration and invasion, resulting in a significant decrease in ECM-degrading urokinase levels but not in MMP-2 levels. An in vivo study showed that hypericin has an antimetastatic effect, only in the dark [169].

The effect of PDT using the second-generation PS Foscan was studied on the expression of various factors that induce head and neck cancer [170]. This study suggested that the decrease in MMP-9 expression was due to inhibition of granulocyte–macrophage colony-stimulating factor, which tends to reduce the binding activity of NF-kB and AP-1 transcription factors. Notably, the cell lines responded differently to PDT. The study did not confirm that PDT decreases the invasive potential of tumor cells in vitro. However, PDT suppressed the activity of three MMPs (MMP-2, -9, and -13), uPA, and VEGF, at least in some oral cancer cell lines [171]. These findings suggest that PDT affects tumor cell invasion and metastasis, potentially assisting in predicting treatment outcomes. Another PS, pyropheophorbide-α methyl ester (MPPa), derived from chlorophyll-α, was found to inhibit metastasis in MCF-7 breast cancer cells by downregulating MMP-2 and MMP-9 expression, which are associated with metastasis initiation [27]. MMP-2 and MMP-9 were not detected in malignant cells after PDT on tumor-bearing rats when an MMP-3 inhibitor was used; the PDT-induced expression was blocked.

The main limitations of conventional PDT are achieving targeted delivery of a PS to cancerous tissues and hypoxic regions. Many tumor tissues display hypoxia, which may limit the clinical effectiveness of PDT. In hypoxic zones, cells produce more MMPs to stimulate the growth of new blood vessels. These areas of MMP activity often remain untreated and become sources of subsequent metastases. Combining PDT with other established anticancer approaches could improve treatment effectiveness [172]. The concept of photodynamic molecular beacons (PMB) was first introduced by Zheng et al. [173]. PMB aims to control a PS’s singlet-oxygen generation, thereby boosting PDT activity. The PMB features a disease-specific quencher linker that prevents photoactivity until it binds to its target, in this case, a tumor-associated MMPs. This work describes a synthetic PMB that produces singlet oxygen upon MMP-7 activation and demonstrates MMP-7-promoted photocytotoxicity against cancer cells. In vivo studies indicated that MMP-7-triggered PDT using these PMBs yielded promising results. Here, selectivity depends not only on the delivery of the PS to malignant cells but also on the specific interaction between the PMB and the biomarker. PDT experiments under discussion showed no effect on the human breast cancer cell line (BT20), which lacks MMP-7 expression. However, this reduced the viability of a human nasopharyngeal epidermoid carcinoma expressing MMP-7.

A new nanomaterial-based PDT combines enzyme-responsive components, suggesting a potential approach for innovative PS delivery in an oxygen-rich environment [174]. The so-called nanoenz-PDT relies on the catalytic action of tumor-related or other external enzymes, which facilitate rapid PS release and subsequent uptake. This process is regulated by the native enzymes surrounding the tumor. Oxygen production mainly depends on catalase-loaded nanoparticles. Using PDT as an adjunct to chemotherapy is a superior treatment, providing a rapid and effective response with minimal side effects and improved patient survival.

The development of an MMP-2-activatable, tunable nanosystem to improve chemo-photodynamic immunotherapy was recently reported [175]. This nanosystem consists of biodegradable mesoporous silica particles loaded with paclitaxel and chlorin e6 (Photolon), conjugated to anti-PD-L1 antibodies via maleimide linkages, and externally coated with gelatin. Upon tumor uptake, MMP-2 degrades the gelatin layer, allowing Ce6 and paclitaxel to enter tumor cells and initiate PDT. Experimental results demonstrated that this nanosystem enhances photosensitizer delivery, cellular uptake, phototoxicity, and immune activation. This study indicates that targeting MMP-2 with PDT could boost the effectiveness of anticancer immunotherapy.

A pilot study of PDT with Ce6 and cisplatin chemotherapy showed significant effects on tumor ECM remodeling [176]. This clinically significant response demonstrates the potential of combining PDT and chemotherapy via local intra-arterial administration of Ce6 and cisplatin for the treatment of HNSCC. Reduced cell migration and invasion, along with downregulation of MMP-2 and MMP-9, were mediated by ROS. According to another study, a “dual-lock” approach that reduces overall effects and improves tumor selectivity requires overexpression of both MMP-2 and cathepsin B enzymes for effective activation [177]. Disabling the ROS-quenching mechanism of Förster resonance energy transfer (FRET) required dual enzyme cleavage to unquench PMB fully. In vitro and buffer-solution tests of MMP-2 responsiveness showed that PMB was more phototoxic to MMP-2-positive cancer cells than to MMP-2-negative cells. MMP-2-positive cells treated with an MMP-2 inhibitor and a cathepsin B inhibitor were used to evaluate the dual-locking effect. Compared to treatment with dual-locked PMB and any enzyme inhibitor, the study in an A549 mouse model found that only dual-locked PMB, combined with PDT, produced a significant antitumor effect. Using an in vivo model, the high selectivity of extrinsic enzyme-activated PS was demonstrated in U87-MG tumors [178]. This work outlined a method for activating PS in a lock-and-key manner, involving targeted delivery to cancer cells and enzyme modification. Compared with traditional activation via intrinsic enzymes, this study proposed an extrinsic enzyme-activatable strategy to reduce nonspecific activation for PDT precision. The drug was activated in the tumor, destroying malignant cells upon laser irradiation, but in another case, only partial reduction was achieved.

Mechanistically, PDT, as a local, mildly invasive method, cannot achieve complete inhibition, and metastasis may still occur. Recently, accumulating evidence indicates that PDT is associated with immunotherapy through the induction of immunogenic cell death [179]. The study suggests that failing tumor cells support immunogenicity and immune cell activation. On the other hand, the enhanced immune response is often limited by the tumor microenvironment. The so-called immune-PD can stimulate the immune response, transforming immune-OFF tumors into immune-ON tumors, thereby achieving a systemic immune response and preventing cancer recurrence [180]. The “double-locked” mechanism can be activated in tumors, and, following laser irradiation, it effectively inhibits tumor growth in vivo without notable side effects. A common side effect of PDT photosensitivity is not mentioned. Overall, these findings demonstrate that the presence of two tumor-associated enzymes may significantly improve selectivity.

Table 2 emphasizes the complexity of PDT’s role in oncology. While PDT primarily aims to affect tumor cells directly, its secondary effect on the TME via modulation of MMPs is dualistic. If not precisely calibrated, PDT could theoretically enhance the invasive potential of surviving cells by stimulating MMPs or crosslinking ECM proteins, thereby making the ECM more resistant to MMP-mediated degradation and suppressing tumor cell migration. To ensure an anti-metastatic effect, it is therefore essential to optimize the PS parameters and light dosimetry. PDT targets highlight the need for further development to enhance selectivity and safety while minimizing photosensitivity in skin and normal tissues. The table summarizes numerous scientific studies investigating how different PSs, when combined with specific light parameters, influence MMPs expression in both in vitro and in vivo cancer models. The data included precursors such as 5-ALA and its methyl ester, which result in the endogenous production of Protoporphyrin IX, and PSs such as Photofrin, Hematoporphyrin derivatives, Chlorins, and Foscan (mTHPC). The impact of PDT with natural PSs, namely curcumin, hypericin, and methylene blue (MB), on MPPs is also described. The most important insight is that MMPs’ response to PDT varies widely across cell line types and treatment conditions. Overexpression of MMP-1, -2, or -9 was observed in several experiments (e.g., those involving Photofrin or 5-ALA). This indicates that activating these enzymes may unintentionally encourage tissue remodeling or possibly tumor cell migration. On the other hand, PSs like zinc phthalocyanine (ZnPc), curcumin, and chlorin e6 frequently cause a substantial decrease in MMP expression. Since the last may prevent tumor invasion and metastasis, this is the intended therapeutic result. Given that biological responses are often dose-dependent, the table highlights the importance of the light dose, measured in J/cm^2^. This leads to either increased tumor cell invasion due to basement membrane degradation or tissue renewal via collagen remodeling. MMPs may be secreted as a survival or repair strategy when low doses cause a cellular stress response. Substantial ROS generation and direct cytotoxicity are usually the result of high doses, which can photodamage cells and impair MMP-enzyme activity. MMP-2 and MMP-9 activities significantly decreased following PDT and showed an inverse correlation with increasing ZnPc dose in irradiated ZR-75-1 cells [181]. The sources of MMPs are far more varied in in vivo models than in in vitro models. Along with tumor cells, endothelial cells drawn by the inflammatory response following PDT and invading immune cells (neutrophils, macrophages) release a significant portion of MMPs.

## 5. Clinical PDT Related to MMPs

Research studies show that members of the MMPs linked to cancer could be proper targets for inhibiting tumor growth and metastases. These findings are accepted as a key to more accurate and effective early cancer detection and curative regimes. The current broad-spectrum small-molecule inhibitors, which have been the main focus towards MMPs for many years, still raise many problems about their lack of specificity, potential side effects, and how to limit these disadvantages in clinical practice. Present efforts focus on developing specific, targeted inhibitors for individual MMPs or groups of MMPs, rather than blocking multiple MMPs simultaneously, a promising approach to improving clinical outcomes. The clinical applications of MMPIs are still in the experimental stage.

A primary study on PDT and MMPs focused on the pathways by which PDT can modulate these proteases to improve cancer treatment results. The PDT approach to MMP behavior offers a major front line in oncology accuracy. Clinical model research indicates that Photofrin-PDT can inhibit tumor progression [186]. It was suggested that a cascade of events is initiated, leading to a temporary increase in the activity of one protease (MMP-9). This requires adjunctive treatment with a different mechanism to prevent accidental enzyme activation, which may be triggered by proper drug delivery. A derivative of 5-aminolevulinic acid, namely methyl aminolevulinate (MAL), has been shown to have effective outcomes in humans with cutaneous excisional wounds [187]. It was observed that PDT-MAL induced the production of MMP-1, MMP-9, and TGF-β3, with their levels further increasing during matrix remodeling, possibly during scar formation, thereby improving the dermal matrix. The potential modulation via the production of transforming growth factor (TGF)-β isoforms was evaluated in patients. TGF-β2 and MMP-1 levels were elevated. MMPs have been assessed in PDT applications that are relevant to oral cavity and skin cancers. Research has identified MB, a phenothiazine compound, as a PDT agent (MB-PDT) for treating oral squamous cell carcinoma (OSCC) and its precursors [188]. The potential of using MMPs as biomarkers of MB-mediated PDT was documented. Since MMP-9 levels are critical for tumor invasion and lymph node metastasis in head and neck cancers, their downregulation serves as a direct biomarker of PDT accuracy. Overexpression of MMP-9 was used to diagnose oral cancer, which is a biomarker in clinical diagnosis. Consistent with the function of MMPs in degrading the basement membrane, higher protein levels of MMP-1, MMP-2, MMP-3, and MMP-9 have been observed in OSCC. Also, the MB-PDT protocol was proposed for the treatment of head and neck carcinoma. This early report suggested that future investigations should compare PDT with another treatment (surgery) in human carcinomas. The elucidation of the molecular pathway by which PDT reduces MMPs in preneoplastic oral cavity and cutaneous cells remains controversial, depending on PDT treatment protocols and the nature of the target cancer cells.

A significant obstacle to successful clinical translation is the poor predictive capacity of current experimental models, despite significant advances in studies combining PDT with MMP targeting. Most in vitro tests cannot reliably predict therapeutic efficacy, immune responses, tumor heterogeneity, or processes observed in patients because they fail to replicate dynamic aspects of the TME, such as fluid flow, mechanical stress, nutrient gradients, and cellular uptake. The main drawbacks of in vivo experiments include physiological differences between humans and animal models, high costs, lengthy durations, and ethical considerations. This gap has been addressed by sophisticated platforms such as organ-on-a-chip systems, which enable the integration of various cell types, immunological components, physiologically relevant mechanical and pharmacological stimuli.

One of the most important factors affecting PDT’s long-term effectiveness is tumor heterogeneity, which frequently leads to recurrence. Different cell subpopulations respond to PSs in different ways (Table 2). There is substantial heterogeneity between tissues and even within tissues, with many different compounds influencing light scattering and absorption. While water may absorb light at longer wavelengths, endogenous chromophores such as hemoglobin can limit efficacy at shorter visible wavelengths. This restricts the range of wavelengths between 600 and 1300 nm that can most effectively penetrate tissue. Nevertheless, light with a wavelength greater than 850 nm lacks the energy required to produce singlet oxygen and activate the PS to its triplet form. To achieve optimal tissue penetration and PS activation, the “therapeutic window” for most PDT applications lies in the red portion of the spectrum between 620 and 850 nm. For instance, melanoma is generally more resistant to PDT because of the presence of the photoprotective pigment melanin, which absorbs light and acts as an antioxidant [50].

Receptor expression in breast cancer varies both within a single tumor and by kind of tumor. Human growth factor receptor 2, progesterone receptor, and estrogen receptor expression are all compromised in triple-negative breast cancer (TNBC), a very aggressive kind of breast cancer. It contains a high concentration of cancer stem cells (CSCs), which are highly resistant to oxidative stress induced by PDT and produce large amounts of MMP-2 and MMP-9. Numerous approaches to eradicating CSC subpopulations have been investigated, as reported in the study by Doustvandi et al. [189]. PDT is a strong and controllable anti-cancer therapy that could eliminate these tumor subpopulations. (ZnPc-PDT at 24 J/cm2 may be a more effective therapeutic option than ZnPc-PDT with 8 J/cm^2^.) However, further investigations into the precise mechanisms of ZnPc-PDT in different cancers are still needed.

## 6. Conclusions, Challenges, and Perspectives

Research on cancer-related MMPs activated during cancer progression supports the development of new anticancer inhibitors and therapeutic strategies. PDT is actively studied as a tool to control MMP expression in inflamed tissues and to suppress it in early cancers, generally showing positive early-stage or adjunctive results in modulating these enzymes’ activity. PDT interactions with cancer-related proteases are highly complex and depend on several internal and external factors. These include tumor type and stage, the characteristics of a particular photoactive compound, the timing of the procedure, the light dose, and the method of administration. The latest evidence shows that PDT can influence MMPs, with both beneficial and harmful effects on cancer. The overall impact is highly complex and dependent, requiring further detailed research to fully understand MMP activity during PDT, both individually and in combination with new inhibitors, to achieve optimal therapeutic outcomes. Much more effort is needed to understand the limitations of the clinical efficacy of MMP upregulation. Pharmacological targeting of MMPs, which play roles in cancer growth and spread, remains a promising research area for developing novel, selective MMP inhibitors, including PDT with new-generation photoactive compounds. Complex drugs that target specific sites could increase inhibitory selectivity. Additionally, side effects associated with PDT, such as the need for adequate oxygen levels in cancer cells, could be mitigated by using specific small-molecule inhibitors to address this limitation. The last approach might also address the selectivity problem and the extended skin photosensitivity of clinically approved PDT drugs used in combination with other treatments.

Future research must be conducted to identify tumor types associated with specific oncogene amplifications and to examine the MMP family to which they belong. A major challenge for new inhibitors will be achieving selectivity at the active sites of MMPs, so they can target individual enzymes without affecting their inactive forms. These findings could help develop new strategies to inhibit proteolytic activity, reduce ECM breakdown, and prevent cancer cell invasion and metastasis. MMP inhibitors have not yet been approved for clinical use. There are several reasons for the limited effectiveness across various cancer types that can be linked to unfavorable clinical applications. Early MMPIs were observed to cause musculoskeletal syndrome, which restricted the maximum tolerated dose for an effective treatment. Administering a single inhibitor drug to patients with advanced, metastatic cancer must be done at the right time. Moreover, the non-specificity of these inhibitors has limited their therapeutic effectiveness, partly because they target a broad range of MMPs and influence the physiological processes. MMPIs may, in fact, be more effective at the beginning of cancer development progression rather than when the cancer has progressed, as well as with all other conventional therapies. In addition, the early stage of MMPI application may be more supportive of general drug toxicity, allowing the administration of a lower dose.

Briefly, PDT is a local cancer treatment, but its efficacy is often limited by the cancer-related MMPs, which are involved in initiating tumor growth and metastasis. One possible way to overcome this limitation is to combine PDT with novel chemical inhibitors and nanotechnologies, yielding a promising synergistic strategy to prevent cancer progression promptly. The future of effective treatment is evident in the shift away from traditional small-molecule inhibitors toward novel targeting approaches to bridge the gap between preclinical success and clinical efficacy. The progress of PDT related to MMP-targeted strategies represents a paradigm shift in oncology, from broad-spectrum treatments to high-precision, enzyme-activated therapy. MMPs tend to serve as both targets and activators during PDT. Novel MMP-activatable nanoplatforms address limitations of traditional PDT, such as non-specific delivery and hypoxia, while reducing damage to healthy tissue and enhancing the destruction of malignant cells. The contradictory effect stems from the highly dose-dependent and cell-specific expression of MMPs during PDT.

## Figures and Tables

**Table 1:** MMPs in cancer.

Classes MMPs		Common Names	Roles in Cancer and Metastases
Collagenases	MMP-1	Interstitial Fibroblast Collagenase-1	Melanoma [48,49,50]; metastasis [51]; bone [52], brain [53], lung [54]; breast cancer in vitro [55]; colorectal cancer [56,57].
	MMP-8	Collagenase-2, Neutrophil Collagenase	Suppresses tumorigenesis and lung metastases in vivo [58]; protective in tongue cancer [59]; in breast carcinoma [60], lung [61], gastric [62], and colorectal cancers [63,64]; and in head and neck squamous carcinoma [65,66].
	MMP-13	Collagenase-3	Overexpressed in invasive phenotype of melanoma [49]; promote bone destruction in vivo [67]; growth, invasion, and angiogenesis of skin squamous cell carcinoma [68]; metastasis of breast cancer [69]; in vivo: prostate cancer [70], colorectal cancer [71], head, neck cancer [72]; non-small cell lung cancer [73]; metastasis of papillary thyroid carcinoma [74], gastric cancer [75].
Gelatinases	MMP-2	Gelatinase A, or Neutrophil Gelatinase, Type IV Collagenase	Breast cancer size [76]; metastasis in melanoma [49]; colorectal cancer worse outcomes [77]; the invasion of breast cancer (in vitro); tumor growth and metastases (in vivo) [77]; brain metastases [78]; stromal (fibroblasts) MMP-2 promote lung metastases (in vitro) [79]; low expression in prostate cancer [80].
	MMP-9	Gelatinase B, Type IV Collagenase	Angiogenesis in melanoma [49]; metastasis, and poor prognosis in cervical [81], colorectal [82] and ovarian cancer [83]; gastric carcinoma with metastatic potential, lymphatic invasion, low survival [84]; tumor vascularization; tumor growth in basal-like triple negative breast cancer [85]; lung metastases [86]; suppress breast cancer (in vivo) [87].
Stromelysins	MMP-3	Stromelysin-1, Transin-1	Melanoma [49]; lung metastases in melanoma [88]; suppress tumor formation (in vivo) [89]; pancreatic adenocarcinoma [90]; protective role during breast cancer development by inhibiting primary and metastatic tumor growth; initial primary tumor formation [91].
	MMP-10	Stromelysin-2	Invasion of head and neck cancer [92]; tongue cancer tissues had more MMP10 expression than normal epithelium [93]; benign prostate tissue [94]; lung cancer stem cells, tumor with metastatic potential [94].
	MMP-11	Stromelysin-3	Tumoral MMP-11 increases the survival of breast cancer cells (in vitro) [95]; Cancer-associated fibroblast-derived MMP11 promotes tumor progression in pancreatic cancer [96].
Matrilysins	MMP-7	Matrilysin-1, PUMP-1, Uterine MP	Colorectal cancer correlates with worse outcomes [97]; increased invasion in gastrointestinal cancers [57];
	MMP-26	Matrilysin-2, Endometase	Prostate cancer in vitro [98]; role in early skin carcinogenesis [99]; pancreatic adenocarcinoma [100].
Transmembrane Type 1	MMP-14	MT1-MMP	Activation of MMP-2 and tumor angiogenesis in melanoma [49]; adverse outcomes in patients with various cancers, including melanoma [101]; promotes tumor growth and angiogenesis in breast cells [102].
	MMP-15	MT2-MMP	Increased expression of MMP-15 in malignant tissue compared to benign prostate tissue [103].
	MMP-16	MT3-MMP	Metastatic prostate cancer tissues compared with nonmetastatic prostate cancer tissues, advanced prostate tumor stage [104]; cell migration and invasion in colorectal cancer [105], melanoma [106], and glioma cancer [107],
	MMP-24	MT5-MMP	Elevated levels have been observed in human brain tumors compared to normal brain tissue [108].
Transmembrane Type 2	MMP-23		Significantly decreased expression in prostate cancer specimens [80]; in melanoma, MMP-23 expression was associated with shorter progression-free survival in patients receiving immune biologics [109].
GPI-anchored	MMP-17	MT4-MMP	Prostate carcinomas, oral carcinomas, osteosarcomas, gastrointestinal adenocarcinomas, leukemias, lung carcinomas, glioblastomas, cervical carcinomas, melanomas, adrenal adeno-carcinomas, thyroid carcinomas [110,111,112,113,114], breast cancer [115], colorectal, head, neck cancer, metastatic phenotype [116].
	MMP-25	MT6-MMP, Leukolysin	Elevated levels of MT6-MMP in human brain tumors compared to normal brain tissue [108]; human colon cancer promotes tumor growth (in vitro) [69]; high expression in malignant versus prostate tissue [103].
Elastases	MMP-12	Macrophage Elastase	MMP-12 expression in colorectal cancer appears to be protective [57]; in human breast cancer [117], tumoral MMP-12 suppresses angiogenesis and tumor growth (in vivo and in vitro) [118].
Other MMPs	MMP-19	RASI-1	Promotes metastatic behavior in vitro and is associated with increased mortality in non-small cell lung cancer [119]; upregulated during melanoma progression and increases invasion of melanoma cells [120].
	MMP-20	Enamelysin	Under pathological conditions, MMP-20 was identified in odontogenic [121] and oral tumors [122], esophageal cancer [123], and human tongue carcinoma cells [124].
	MMP-21		High expression in pancreatic adenocarcinoma [100]. A low survival in humans with colorectal cancer [125].
	MMP-27		tumor suppressor [126], decreased MMP-27 is associated with poor differentiation and increased tumor invasiveness, particularly bone invasion, in oral squamous cell carcinoma [127].
	MMP-28	Epilysin	The roles of epilysin in cancer appear to vary by tumor type and disease stage [128].

**Table 2:** **PDT regimes that influence MMPs.** The effects of type photosensitizers, light parameters, and reactions on the targeted cancer cells or cancer models, while also having nonspecific effects on MMPs.

Photosensitizer	Light Parameters	In Vivo Cancer Model	In Vitro Cancer Cells	Influence on MMP
*Methyl ester* of *5-ALA* *aminolevulinic acid* and 5-*ALA* [166]	In vitro: 630 nm, 10 J/cm^2^, 2, 6, or 24 h incubation; 100 mW/cm^2^ In vivo: Halogen lamp at 630 +/− 20 nm, 100 J/cm^2^ (24 h post-injection)	Mammary gland, rat	LoVo, a colon adenocarcinoma cell line, and MCF-7, a breast cancer cell line.	In vitro: showed that MMP-2 and MMP-9 are not induced after PDT. MMP-3 was induced and expressed in the tumor both in vitro and in vivo. In vivo, MMP-3 expression was mainly observed in the tumor and skin after PDT.
*Photofrin* [167]	In vitro: 0.35 mW/cm^2^, 10 min In vivo: 75 mW/cm^2^; up to 200 J/cm^2^	BA tumor, C3H/HeJ mice, 8–12-week-old female	Mouse mammary carcinoma (BA) cells	MMP-1, -3, and -8 in tumor tissue after PDT. MMP-9 levels increased in the supernatant, whereas expression in tumor cells was low.
*Hypericin* [169]	In vitro: A wide-band illumination with 585 nm, 0.5 J/cm^2,^ 2 min In vivo: 14 J/cm^2^ and 27 mW/cm^2^	HK1 tumor, BALB/c nude mice of 6–8 weeks old	HK1 NPC cells from an NPC patient diagnosed with recurring well-differentiated squamous cancer	In vitro: the total MMP-9 content in cell culture supernatant was reduced in HK1 cells. Downregulation of MMP-9 transcription in HK1 cells, with mRNA reduced by 76% at 1 h post-PDT. In vivo: MMP-9 transcription was also downregulated in HK1 tumor tissues.
*Hematoporphyrin monomethyl ether* [182]	630 nm (DIOMED 630 nm), 80 J/cm^2^, 10 min	Rat C6 glioma model		MMP-2 expression decreased.
*Meta-tetrahydroxyphenylchlorin (mTHPC)* *Meso-tetraphenylporphyrin disulfonate (TPPS_2_)* *Disulfonated aluminum phthalocyanine (AlPcS_2_)* [165]	652 nm, diode laser, 100 mW/cm^2^ 430 nm (390–450 nm), fluorescent tubes blue light; 7 mW/cm^2^ 635 ± 5 nm, LED; 55 mW/cm^2^, 14 min		Human gingival cancer (oral squamous cell carcinoma, Ca9-22)	Increase in MMP-10 mRNA
*9-Hydroxypheophorbide alpha-chlorophyll* derived *PS* [172]	664 nm diode laser at an energy density of 2.0 J/cm^2^ for 15 min		The laryngeal squamous carcinoma Hep-2 cell line.	Downregulated expression of MMP-2 and MMP-9
*Pyropheophorbide-α methyl ester (MPPa)* [176] *Pyropheophorbide-α methyl ester (MPPa)* [27]	630 nm; continuous mode; 40 mW/cm^2^, 4.8 J/cm^2^ 630 nm (30 mW/cm^2^) for 30, 60, 90, 120, or 180 s to obtain an energy density of 0.9, 1.8, 2.7, 3.6, or 5.4 J/cm^2^		Osteosarcoma cell line (MG-63) MCF-7 breast cancer cells.	MMP-2 and -9 expression decreased Downregulated the expression of MMP-2 and MMP-9
*Meta-tetrahydroxyphenyl chlorin (m-THPC*; *temoporfin*; *Foscan1)* [171]	652 nm diode red laser light with 25 mW/cm^2^; 0.25–4 J/cm^2^		Oral squamous cell carcinomas (OSCC), H37620, VB621, and UP	Both active and latent MMP-2 and MMP-9 were downregulated by UP and VB6 cells, while H376 showed an increase in active-MMP-2
*Methylene blue (MB)* [182]	664 nm DD4, up to 10 J/cm^2^ (6.3–6.5 J/cm^2^) and 16–38 mW/cm^2^, 32 mW/cm^2^		Oral squamous cell carcinoma (CA-9-22), oral leukoplakia cells (MSK-Leuk1)	MMP-9 gene expression was found to be significantly decreased in oral carcinoma and leukoplakia
*5-ALA* and *ALA hexylester (ALA-H)* [166]	630 nm, 4 J/cm^2^		Medulloblastoma (MED) cell line (TE-671)	MMP-2 expression is down-regulated. The MMP-9 expression remains unchanged after treatment.
*Zinc phthalocyanine* [183]	675 nm GaAlAs laser (70 mW), 15 J/cm^2^, 1 min.		Human breast cancer cell line (ZR-75-1)	Significant decrease in the activity of MMP-2 and MMP-9 after PDT. By increasing the dose of ZnPc in irradiated ZR-75-1 cells, a reverse correlation was observed for MMP-2 and MMP-9.
*Pheophorbide a* [184]	664 nm laser, 2.0 J/cm^2^ for 15 min		Human prostate cancer cells (PC-3)	Downregulate the expression of MMP-2 and MMP-9 in PC-3 cells
*Chlorin e6*—*PVP (Fotolon OAO)* [50]	662 laser, 5 J/cm^2^, 10 J/cm^2^ and 20 J/cm^2^		Skin melanotic type line (Colo-829) and metastatic line from pleural effusion (SH4)	Inhibition of expression of the MMP-2, MMP-9, and MMP-14
*Curcumin* [185]	Blue LED light (365 nm) at time intervals of 5, 10, 15, 20, and 25 min.		Human glioblastoma (T98G) cells	Expression of MMP-2 and MMP-9 decrease

## Data Availability

No new data were created or analyzed in this study. Data sharing is not applicable to this article.

## Abbreviations

MMPIs	Matrix Metalloproteases Inhibitors
TAM	Tumor-Associated Macrophages
MMPs	Matrix Metalloproteases
PDT	Photodynamic Therapy
ECM	Extracellular Matrix
PS	Photosensitizers
